# Supplementary material for: Non-Pharmacological Interventions for Type 2 Diabetes in People Living with Severe Mental Illness: Results of a Systematic Review and Meta-Analysis
Source: Int J Environ Res Public Health. 2024 Mar 30;21(4):423. doi: 10.3390/ijerph21040423 (PMC11049919; doi:10.3390/ijerph21040423)
Supplement: Supplementary file 1 [file ijerph-21-00423-s001.zip › ijerph-2923218-supplementary.pdf]

Non-pharmacological interventions for type 2 diabetes in people living with severe mental illness: Results of a systematic review and meta-analysis – Supplementary Tables

Table S1. Search syntax

Search within	*Search terms*
ABS-TITLE-KEYWORDS	schizophrenia OR schizoaffective OR "schizoaffective disorder" OR bipolar OR "bipolar disorder" OR psychosis OR "major depress*" OR "SMI" OR "severe mental illness" OR "severe mental disease" OR "severe mental disorder"
AND	
TITLE	diabetes OR "diabetes mellitus" OR "diabetes mellitus type 2" OR "diabetes type 2" OR "type 2 diabetes"
AND	
ABS-TITLE-KEYWORDS	non-pharmacological OR lifestyle OR exercise OR physical OR diet* OR nutrition* OR psycho* OR cognitive OR behaviour* OR intervention OR therapy OR activity OR trial OR management
No time or language restriction applied.	

Table S2. Eligibility criteria

	**Include**	**Exclude**
Patient problem / Population	Patients with types 2 diabetes and SMI;<br><br>18 years and over	Pre-diabetes, Gestational diabetes, people without diabetes, patients with type 1 diabetes<br><br>Patients with mental health problems other than SMI: e.g., depression, anxiety, eating disorder, smoking and alcohol abuse etc.
Intervention	Non-pharmacological interventions targeted at diabetes management in people with SMI – Diet, exercise, other lifestyle, educational and behavioural interventions, and/or change to care delivery.	Medication intervention, mixed interventions including new medication in combination with lifestyle/behaviour interventions
Comparison or control	Studies involving a comparator (usual care/practice, medication,	Case studies

	other non-pharmacological intervention or waiting list) or studies with multiple time points of data in the same study population	
Outcomes	• Primary outcomes:<br>• Glycaemic Control: Glycated haemoglobin, fasting blood glucose<br>• Psychiatric symptoms<br>• Quality of Life (QoL)<br><br>• Secondary outcomes:<br><br>• Lipid profile – LDL, HDL, Triglycerides, Total Cholesterol<br>• Economic Cost<br>• Body mass index<br>• Hospital admissions	
